# Short-Lived, Transitory Cell-Cell Interactions Foster Migration-Dependent Aggregation

**DOI:** 10.1371/journal.pone.0043237

**Published:** 2012-08-17

**Authors:** Melissa D. Pope, Anand R. Asthagiri

**Affiliations:** 1 Department of Chemical and Biological Engineering, University of Colorado at Boulder, Boulder, Colorado, United States of America; 2 Department of Chemical Engineering, Northeastern University, Boston. Massachusetts, United States of America; Northwestern University Feinberg School of Medicine, United States of America

## Abstract

During embryonic development, motile cells aggregate into cohesive groups, which give rise to tissues and organs. The role of cell migration in regulating aggregation is unclear. The current paradigm for aggregation is based on an equilibrium model of differential cell adhesivity to neighboring cells versus the underlying substratum. In many biological contexts, however, dynamics is critical. Here, we provide evidence that multicellular aggregation dynamics involves both local adhesive interactions and transport by cell migration. Using time-lapse video microscopy, we quantified the duration of cell-cell contacts among migrating cells that collided and adhered to another cell. This lifetime of cell-cell interactions exhibited a monotonic decreasing dependence on substratum adhesivity. Parallel quantitative measurements of cell migration speed revealed that across the tested range of adhesive substrata, the mean time needed for cells to migrate and encounter another cell was greater than the mean adhesion lifetime, suggesting that aggregation dynamics may depend on cell motility instead of the local differential adhesivity of cells. Consistent with this hypothesis, aggregate size exhibited a biphasic dependence on substratum adhesivity, matching the trend we observed for cell migration speed. Our findings suggest a new role for cell motility, alongside differential adhesion, in regulating developmental aggregation events and motivate new design principles for tuning aggregation dynamics in tissue engineering applications.

## Introduction

Multicellular aggregation is fundamental to embryonic development and tissue repair [1]. In the early stages of limb development, for example, aggregation of cartilage precursor cells (chondrocytes) is a prerequisite for cellular differentiation [2]. Multicellular aggregation also plays a role in heart development: cells delaminate from the atrioventricular canal and re-assemble to form the heart valves [3]. De-regulation of multicellular aggregation functions in pathologies such as metastasis, which is associated with the loss of aggregate integrity [4]. Therefore, understanding the biophysical principles that govern multicellular aggregation will both enhance our understanding of development and disease and contribute design strategies to tune the formation of aggregates in applications such as tissue engineering.

A classical paradigm is that the equilibrium state of aggregation is determined by minimizing the adhesive free energy of the system [5]–[7]. This model predicts that if the cumulative strength of cell-cell adhesion (as quantified by the number and affinity of receptor-ligand bonds) exceeds the strength of cell-substratum adhesion, cells will organize into aggregates. Conversely, if the strength of cell-substratum adhesion exceeds the strength of cell-cell adhesion, cells will adopt a dispersed phenotype. This monotonic relationship between aggregation and substratum adhesivity has been demonstrated experimentally [8]. When cells of equal cohesivity were employed, those seeded onto weakly adhesive substrata aggregated while those seeded onto highly adhesive substrata dissociated.

**Figure 1:**
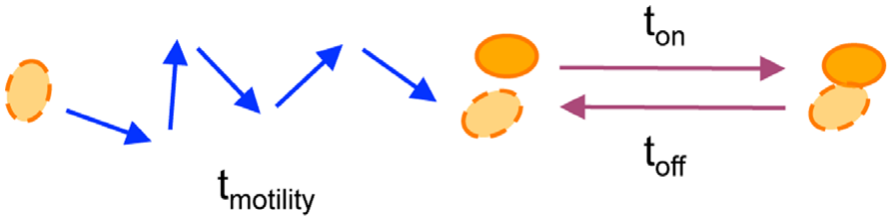
Two-step model for multicellular aggregation dynamics. To form aggregates, distant cells must first move close together (a transport step) and then undertake reversible cell-cell interactions (a reaction step). Transport occurs by cell migration, and the mean time to collide (t_motility_) depends on the mean initial spacing between cells (L_o_) and the speed and persistence of cell movement. Meanwhile, the local cell-cell interaction involves adhesion (t_on_) and detachment (t_off_).

In many biological contexts, however, the *dynamics* of aggregation – not solely the equilibrium state – is likely to be critical. The development of tissues and organs, for example, proceeds through multiple stages, and each step, such as multicellular aggregation, must be accomplished within a defined time window. The current equilibrium model for multicellular aggregation, however, considers only the strength of cell-cell and cell-matrix adhesions. When assessing dynamics, the rate at which cells move to encounter each other will also be an important factor (Figure 1). It is well-established in physicochemical systems ranging from colloids [9] to atmospheric chemistry [10] that aggregation is a two-step process: individual particles must first move and encounter each other (a transport step) and then form stable contacts (a reaction step). Aggregation dynamics is then dictated by the slower of the two steps.

How the interplay between transport and reaction affects aggregation dynamics in cellular systems is unclear. In these systems, the transport step is mediated by cell migration. It is well-documented that cell speed exhibits a biphasic dependence on substratum adhesivity: weakly adhesive substrata do not enable the cell to generate the required traction, while strongly adhesive substrata prevent rear release after the cell body translocates forward [11]. Therefore, if transport is indeed the rate limiting step, aggregation dynamics may exhibit a biphasic dependence on cell-substratum adhesivity that contrasts with the monotonic trend predicted by the classical equilibrium model and reported in experimental studies of cell aggregation that have been performed to-date [5]–[8].

It is currently a challenge to compare the dynamics of transport and reaction for cellular systems. In contrast to the large body of quantitative studies of cell migration [11], to our knowledge, there is currently no evaluation of the timescale on which migrating cells “react” to form intercellular contacts. Although cell-cell contact dynamics has been studied for cells brought together with micropipettes [12], interactions between migrating cells are likely to be significantly different. Migrating cells interact with each other while concomitantly adhering to an underlying substratum. This mode of cell-cell interactions is significantly different from interactions between cells held in suspension or by micropipette aspiration.

Here, we developed and applied a quantitative approach to measuring the lifetime of cell-cell interactions among colliding migrating epithelial cells cultured on a laminin (Ln)-coated substrata. Through parallel quantitative measurements of cell motility and multicellular aggregation dynamics, we explored whether multicellular aggregation dynamics is in fact dictated by the relative timescales of cell-cell adhesion and cell motility, and therefore described by the transport-reaction model that describes physiochemical systems.

## Results

**Figure 2:**
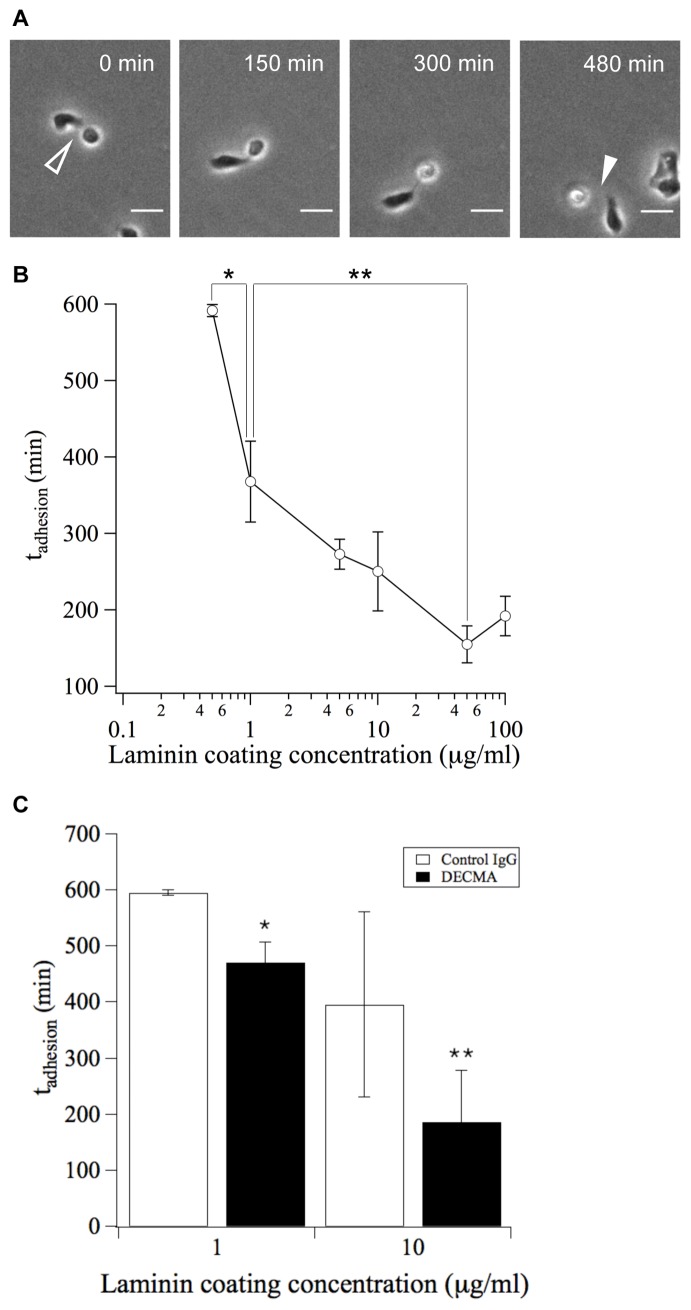
The timescale of local cell-cell reactivity (t_adhesion_) is dependent on substratum adhesivity. (A) Timelapse phase contrast images of migrating MDCK cells show the initiation of cell-cell contact (open arrowhead) and the subsequent detachment (closed arrowhead). Because t_on_ and t_off_ cannot be distinguished experimentally, timelapse images were used to quantify the total duration of cell-cell interactions (t_adhesion_) as a lumped measure of t_on_ and t_off_. Scale bar, 25 µm. (B) The duration of cell-cell interactions (t_adhesion_) was quantified for substrata prepared with different coating concentrations of Ln. Error bars, s.e.m. (n = 2–3 trials; N>50 cell pairs per trial). *P<0.06 between 0.5 µg/mL and 1 µg/mL conditions. **P<0.07 between 1 µg/mL and 50 µg/mL conditions. (C) The duration of cell-cell adhesions (t_adhesion_) was measured in the presence of an E-cadherin-specific or non-specific control IgG. Error bars, s.e.m. (n = 2 trials; N = 30–80 cell pairs per trial). *P<0.05 between DECMA and Control IgG conditions for 1 µg/mL Ln coating concentration. **P<0.2 between DECMA and Control IgG conditions for 10 µg/mL Ln coating concentration.

To quantify the dynamics of cell-cell interactions, we identified cell-cell collisions in time-lapse videos and recorded the duration of intercellular contact (Figure 2A). These measurements were performed using substrata coated with different amounts of the adhesion ligand laminin (Ln) in order to better understand how varying substratum adhesivity affects the lifetime of cell-cell interactions. We observed that the mean lifetime of cell-cell interactions (t_adhesion_) exhibits a monotonic dependence on substratum adhesivity (Figure 2B). Increasing adhesion ligand density reduced the lifetime of cell-cell interactions: t_adhesion_ was nearly 600 min on substrata of low adhesivity and was reduced to approximately 200 min on substrata of high adhesivity.

To confirm that our measurements captured specific cell-cell interactions, we treated cells with an antibody (DECMA) that blocks E-cadherin, a cell surface receptor that mediates intercellular adhesion. Treatment with DECMA reduced t_adhesion_ compared to treatment with a non-specific IgG control, confirming that E-cadherin is involved in mediating these cell-cell interactions (Figure 2C).

To assess how the measured lifetime of cell-cell interactions compares with the timescale of transport, we next examined the migration of individual cells on Ln-coated substrata. Cell migration in an isotropic environment exhibits an unbiased persistent random walk characterized by a diffusivity or motility coefficient (μ) that is related to the speed (S) and directional persistence (P) of cell movement [13], [14]. Migrating cells were tracked using time-lapse video microscopy, and cell speed was determined by fitting mean squared displacements to a persistent random walk model.

**Figure 3:**
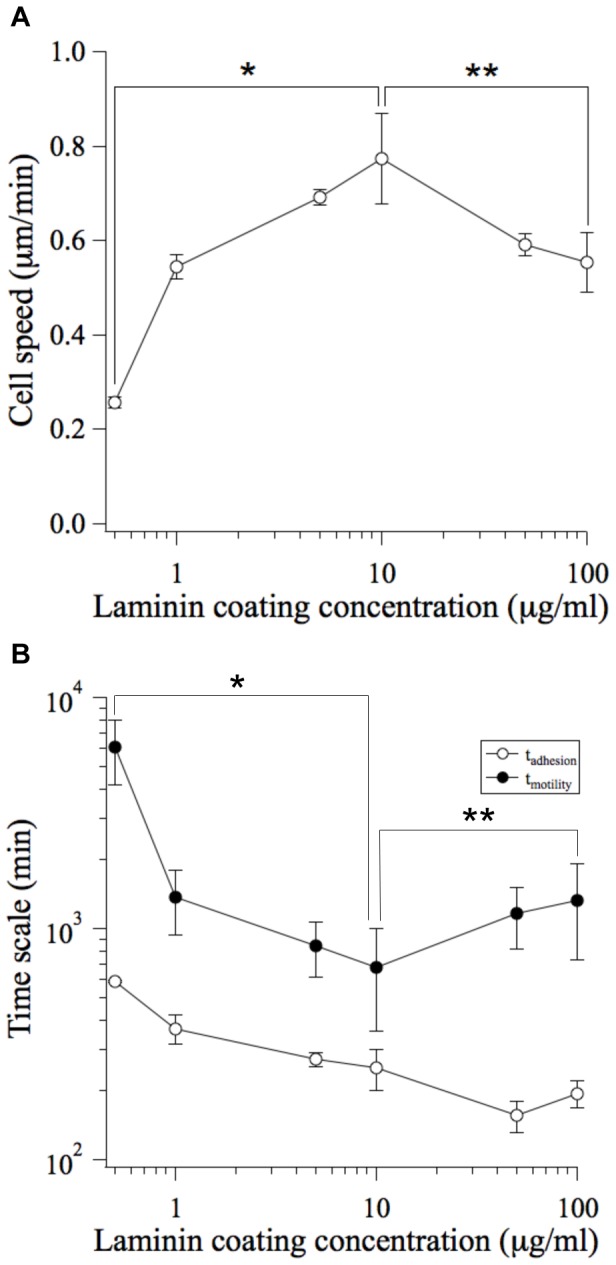
Quantitative comparison of the timescales of cell-cell reactivity (t_adhesion_) and transport (t_motility_). (A) The migration tracks of individual MDCK cells were traced using timelapse microscopy. Mean squared displacements were calculated and fit to a persistent random walk model to determine cell speed [13], [14]. Error bars, s.e.m. (n = 2–3 trials; N>20 cells per trial). *P<0.05 between 0.5 µg/mL Ln and 10 µg/mL Ln conditions. **P<0.1 between 100 µg/mL Ln and 10 µg/mL Ln conditions. (B) The mean time for nearest neighbors to collide (t_motility_) was quantified for substrata prepared with different coating concentrations of Ln. t_adhesion_ is re-plotted here for the purpose of comparison (see Figure 2B). t_adhesion_: error bars, s.e.m. (n = 2–3 trials; N>50 cells per trial). t_motility_: error bars, s.e.m. (n = 2–3 trials; N>20 cells per trial). *P<0.05 between 0.5 µg/mL Ln and 10 µg/mL Ln conditions. **P<0.1 between 100 µg/mL Ln and 10 µg/mL Ln conditions.

Cell speed exhibited the expected biphasic dependence on substratum adhesivity [11]. In our system, the peak cell speed was 0.77±0.09 µm/min on substrata coated with 10 µg/mL Ln (Figure 3A). The measured values of S and P (29.1±6.4 min) were consistent with published values for epithelial cell lines [15]–[17].

Using these values for S and P, we calculated the mean time required for a cell to collide with its nearest neighbor (t_motility_ = L_o_^2^/μ) where L_o_∼108 µm is the mean intercellular spacing based on the initial cell density of 8.5×10^3^ cells/cm^2^ and the motility coefficient μ is equal to S^2^P. This initial cell seeding density was selected because it is sufficiently low to facilitate a starting condition of individual cells, and not pre-formed cell clusters (see Information S1). The calculated time scale for transport exhibited a biphasic dependence on substratum adhesivity; therefore cell-cell collisions were infrequent on substrata of low and high adhesivity (t_motility_ = 6.1±1.8×10^3^ and 1.3±0.6×10^3^ min, respectively) and occured with greatest frequency on substrata of moderate adhesivity (t_motility_  = 6.8±3.2×10^2^ min) (Figure 3B).

Comparing the measured time scales of transport and local reactivity revealed that t_motility_ was greater than t_adhesion_ across the complete range of Ln coating concentrations (Figure 3B). Therefore, if multicellular systems follow the general two-step principle of aggregation dynamics, we would expect aggregation dynamics to be transport-limited and to exhibit the biphasic dependence on substratum adhesivity observed for cell speed.

**Figure 4:**
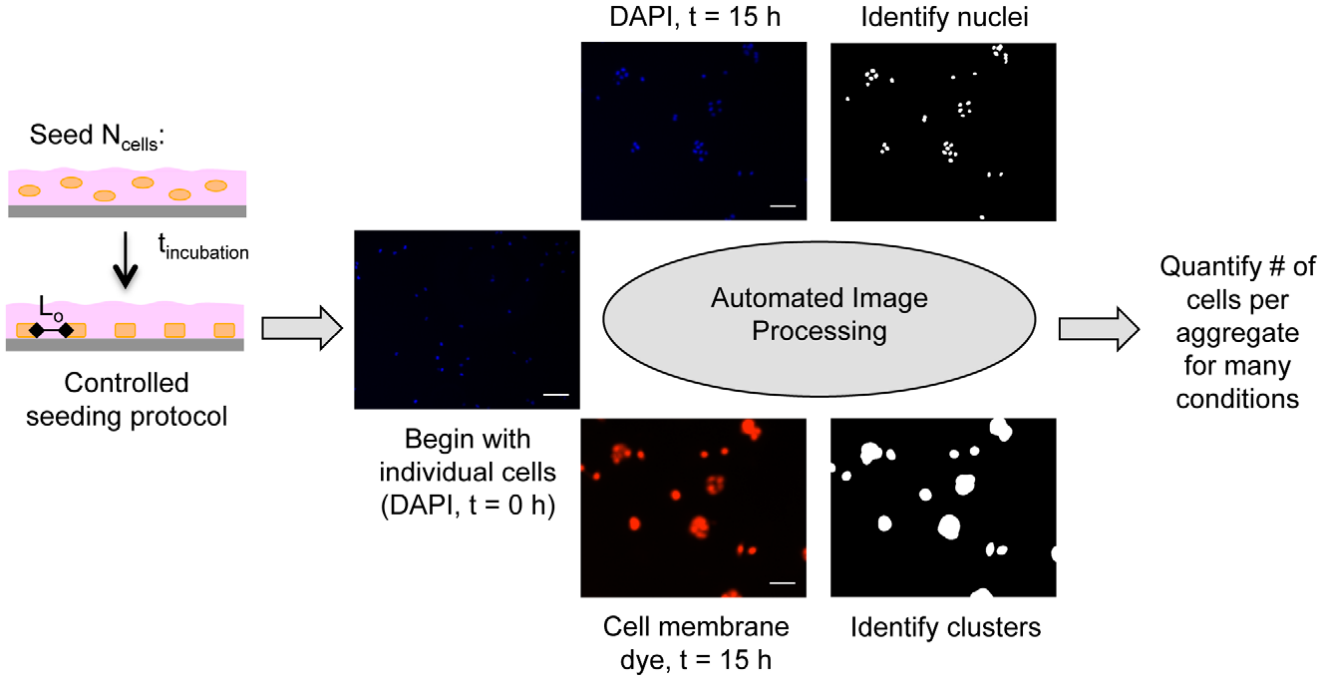
Techniques for quantifying aggregate size. N_cells_ (0.8–2.6×10^4^ cells/cm^2^) were seeded onto Ln-coated substrata and allowed to adhere for t_incubation_ (1–3 hours). Non-adherent cells were then rinsed away such that only individual, substratum-attached cells remained at t = 0 h. N_cells_ and t_incubation_ were optimized for each Ln coating concentration such that the initial mean intercellular spacing (L_o_) was ∼108 µm for all substrata. Aggregate sizes at t = 0 h and t = 15 h were determined from fluorescence images of DAPI-labeled nuclei and membrane dye-labeled clusters using thresholding and edge detection algorithms in MATLAB. The number of nuclei contained within each cluster was determined using MATLAB, providing a rigorous and high throughput method for quantifying aggregate sizes.

Testing this hypothesis, however, was not straightforward. Varying substratum adhesivity affected the number of seeded cells that attached to the substratum, and thereby introduced unwanted differences in L_o_ among substrata (Figure S1). In addition, adequate time needed to be allowed for cells to attach to the substratum – a particularly important concern for substrata of low adhesivity (Figure S2 and Table S2). Furthermore, any non-adherent cells needed to be removed to ensure that the observed multicellular aggregation is the result of collisions between adherent, migrating cells and not between drifting cells in suspension. Finally, cells needed to be plated at a low enough density to avoid pre-clustering of cells in suspension and to ensure an initial condition of isolated cells (Table S3). Guided by these and other considerations, we developed a rigorous protocol for studying the effect of substratum adhesivity on multicellular aggregation dynamics (Figure 4; also see Information S1). Our method yielded highly uniform initial conditions: for all Ln coating concentrations, the initial density of substratum-attached cells was 8.5±0.2×10^3^ #/cm^2^ (Table S1). This density corresponded to an initial mean intercellular spacing of L_o_∼108 µm.

To quantify aggregate sizes, two-channel fluorescence images were acquired of multicellular aggregates labeled with nuclear and cell membrane markers (Figure 4). Nuclei and cell clusters in the images were identified using automated image processing techniques [18], and the mean number of cells per aggregate was determined. Our methods facilitated rigorous, high throughput quantification of aggregate sizes.

**Figure 5:**
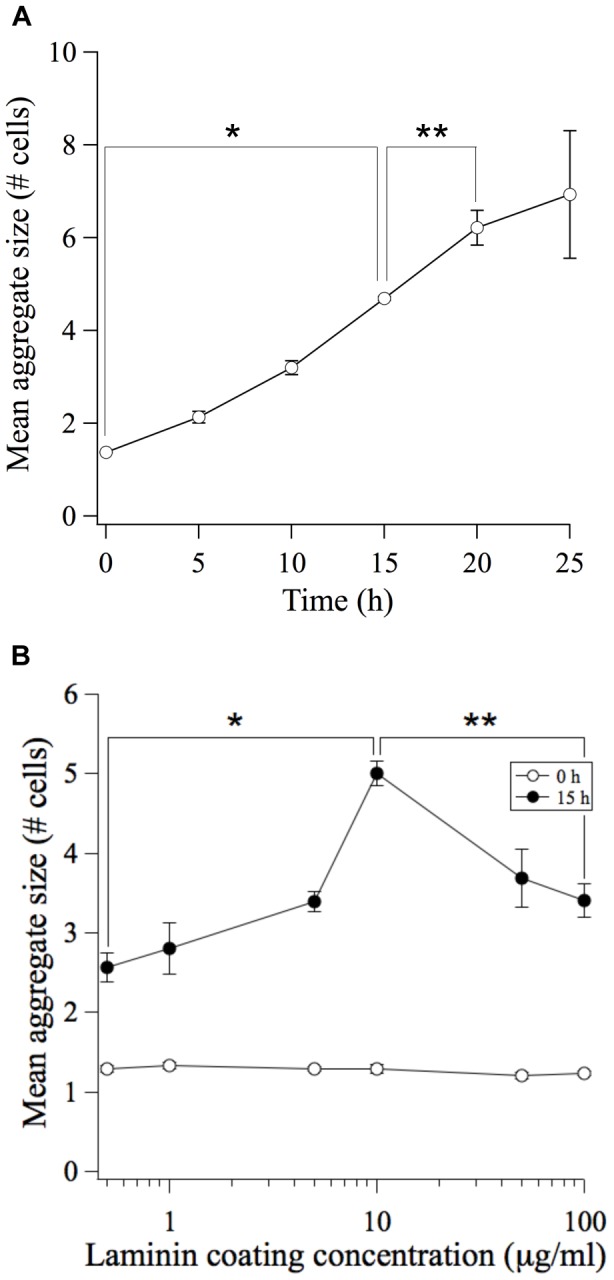
Biphasic dependence of aggregate size on substratum adhesivity: evidence for motility-limited aggregation dynamics. (A) The mean aggregate size (# of cells per aggregate) was quantified at initial time (0 h) and at multiple timepoints after the attachment of MDCK cells to the Ln-coated (10 µg/mL) substratum. Error bars, s.e.m. (n = 2 trials; N>350 aggregates per trial). *P<0.05 between 0 h and 15 h conditions. **P<0.06 between 15 h and 20 h conditions. (B) The mean aggregate size (# cells per aggregate) was quantified at initial time (0 h; open circles) and 15 h (closed circles) after the attachment of MDCK cells to Ln-coated substrata. Error bars, s.e.m. (n = 3–4 trials; N>350 aggregates per trial). *P<0.0005 between 0.5 µg/mL Ln and 10 µg/mL Ln conditions. **P<0.005 between 100 µg/mL Ln and 10 µg/mL Ln conditions.

We first performed a time course study to identify an appropriate time-point at which to examine the dynamics of our system. Mean aggregate size was found to increase monotonically with time with the process continuing to evolve as late as 20 h after cell seeding (Figure 5A). Therefore, we selected 15 h as an appropriate time point at which to capture aggregation during its dynamic phase. During this 15 h time period, we expect that MDCK cells will undergo proliferation. We note, however, that the doubling time for MDCK cells is greater than 15 hours (typically ∼24 h); therefore proliferation is not expected to influence aggregate growth in our experiments. To confirm more directly the magnitude of the proliferation effect, we quantified cell number at the start of the experiment (t = 0 h) and after incubating for 15 h. On substrata coated with 10 µg/mL laminin, we found that cell number increased only 1.6±0.1 fold compared to a 5 fold increase in aggregate size (Figure 5B).

The mean aggregate size at 15 h was quantified for cells seeded on substrata coated with different amounts of Ln. We observed that the mean aggregate size exhibited a biphasic dependence on substratum adhesivity (Figure 5B). Moreover, the maximum aggregate size occurred at the Ln coating concentration of 10 µg/ml, matching the conditions at which cell speed is maximum. These findings were consistent with a two-step physiochemical model for multicellular aggregation dynamics. Furthermore, these data demonstrated that the time scale of local reactivity was sufficiently fast (200–600 min) to render aggregation dynamics transport-limited. The consequence was that aggregation dynamics follows a non-monotonic dependence on substratum adhesivity in contrast to the equilibrium perspective of the differential adhesion paradigm and to the experimental reports of cell aggregation to-date [8].

## Discussion

In this work, we used quantitative analysis of time-lapse microscopy to measure the sensitivity of cell-cell interactions among migrating cells to the modulation of cell-matrix adhesion. We found that the more adhesion ligand coated on a substratum, the shorter the lifetime of cell-cell interactions. Our study revealed that the characteristic time scale of cell-cell interactions ranges between 4–10 h in MDCK cells depending on the amount of Ln used to coat the substratum. This inverse monotonic relationship between cell-cell and cell-matrix adhesions has been suggested by studies of multicellular processes, for example, cell aggregation [8] and scattering [19]. But to our knowledge, this study offers the first direct quantitation of the lifetime of cell-cell interactions among individual migrating cell pairs.

What is the mechanism behind this cross-talk between cell-matrix and cell-cell adhesion? Many of the molecular components mediating cell-substratum adhesions during cell migration are also involved in cell-cell interactions [20], [21]. As a result, integrin engagement by the underlying ECM matrix can cross-regulate cadherin-mediated adhesions and modulate the formation of cell-cell junctions. For example, integrin-mediated activation of Src was shown to destabilize endothelial cell-cell contacts by modulating γ-catenin-VE-cadherin binding [22]. In addition, cell-cell and cell-substratum adhesions engage a common cytoskeleton and are coupled through mechanotransduction and cell-generated contractile forces [20], [21]. Therefore, cross-talk between cell-cell and cell-matrix adhesion can also be a physical response to cell-generated forces. During MDCK cell scattering, for example, integrin-mediated actomyosin contractions at the cell periphery were shown to physically disrupt cadherin-mediated cell-cell adhesions [19].

Our direct measurement of the characteristic time scale of cell-cell reactivity enabled a quantitative comparison of the dynamics of cell-cell adhesion and cell migration. This comparison is instructive from the perspective of gauging the rate-limiting step governing multicelluar aggregation. We hypothesized that if cell migration-mediated transport operated on a slower timescale than this characteristic time of cell-cell reactivity, then aggregation would be migration-limited. We tested and confirmed this hypothesis. The significance of this finding is that when aggregation is transport-limited, it exhibits a biphasic dependence on adhesion ligand density, a trend that contrasts what has been reported in aggregation experiments to-date [8] and differs from the current theoretical paradigm that is largely based on equilibrium models [5]–[7].

Why was the transport-limited, biphasic dependence of aggregation on substratum adhesivity evident in our experiments but not in previously reported work? A primary reason is that the transport step of aggregation in our system was mediated solely by cell migration. Cells were seeded onto the substrata at density low enough to avoid pre-clumping of cells during the settling and attachment process. Furthermore, even at the lowest Ln coating concentrations, we ensured that only adherent cells are left in the system to participate in aggregation. Hence, aggregation could not occur by non-adherent cells clumping together in suspension.

The fact that aggregation dynamics exhibits a more complex dependence on adhesion ligand density than previously understood is important in both physiological and engineering contexts. For tissue engineering applications, engineering biomaterials with reduced adhesivity may enhance aggregation at equilibrium [23]. However, we suggest that a different strategy is needed to control aggregation kinetics. For example, maximizing aggregation kinetics would be of particular importance when working with a cell type that exhibits a survival advantage when clustered [24]–[26]. When dynamics is the chief concern, we propose that the design strategy should be to assess and to account for whether transport or local cell-cell reactivity is rate-limiting. Where transport is rate-limiting, a biomaterial with intermediate adhesivity will likely provide maximal aggregation dynamics. In an adhesion-limited regime, one would expect a linear dependence of aggregate size on substratum adhesivity that mimics the trend observed for t_adhesion_. In this case, engineering biomaterials with reduced adhesivity would likely provide maximal aggregation dynamics.

In addition to substratum adhesivity, cell seeding density is a key design element in tissue engineering applications. Increasing cell seeding density decreases the initial intercellular spacing (L_o_), thereby reducing t_motility_. In fact, modulation of L_o_ has the potential to shift a system from a transport-limited regime to an adhesion-limited regime. For the MDCK system used in this study, it can be predicted from our measurements of t_motility_ and t_adhesion_ that at a L_o_∼33.8 µm (t_motility_ = t_adhesion_ = L_o_^2^/μ), aggregation dynamics would transition from motility-limited to adhesion-limited. This value of L_o_ corresponds to a cell seeding density of 3.0×10^6^ cells/cm^2^. MDCK cells, however, pre-cluster in suspension at such a higher seeding density, rendering it impossible to achieve an initial condition of isolated individual adherent cells. Cells with greater migration speed and/or shorter-lived cell-cell interactions than MDCK cells would provide an effective system to examine the effect of varying cell density on the transition from motility-limited to adhesion-limited aggregation.

The work we present here highlights that multicellular aggregation dynamics is affected by multiple parameters in an interconnected manner. Our transport-reaction model for multicellular aggregation dynamics therefore motivates a need to couple the choice of substratum adhesivity and cell seeding density for tissue engineering designs. Future measurements of the lifetime of cell-cell interactions and cell migration as described here could lead to a phase diagram that places cell types and microenvironment variables, such as the type and amount of adhesion ligand, on a map of adhesion- and motility-driven aggregation dynamics. Such a phase diagram would be a useful guide for synthetic design and provide insight into biological contexts in which adhesion or motility governs aggregation dynamics.

In this work, we modulate adhesion ligand density by varying the coating concentration of the adhesion ligand Ln, then quantify the affect on multicellular phenomena. Such alterations in substratum adhesivity are highly physiologically relevant. It is well-established that adhesion ligand expression is modulated during developmental events, tissue repair and disease. For example, an elevated presence the extracellular matrix protein fibronectin is observed during wound healing [27] and mammary tumorigenesis [28], [29]. Our findings emphasize that these alterations to local adhesivity do not simply mediate a monotonic trend in aggregation, but affect more complex changes to tissue architecture that encompasses both transport and adhesive components.

Our findings also highlight a key, previously unrecognized role for cell motility in multicellular aggregation. Evidence is also mounting for the role of cell motility in mediating another multicellular process: cell sorting of heterogeneous cell populations. Though proposed years ago as a potential mediator of cell sorting, differential motility has only recently been discussed as a driving mechanism for this phenomenon [30], [31]. A recent mathematical model of *Dictyostelium* slug formation, for example, demonstrates that motility differences among cell types are sufficient to create the defined spatial pattern of cells observed in migrating slugs [32]. In addition, cellular rearrangements within epithelial tissues have been attributed to differential motility: cells expressing high levels of the enzyme MMP14, which preferentially localize to the tip of epithelial tubes, were found to be faster and more directionally persistent than their low-expressing counterparts [17]. These studies of cell sorting together with our results pertaining to cellular aggregation demonstrate the emerging importance of a cell motility component in the dynamics of multicellular re-arrangements.

In conclusion, our results provide biophysical insights into *in vivo* aggregation events and a dynamical physical perspective on engineering microenvironments to promote multicellular aggregation, an important precursor to more mature multicellular structures and tissues.

## Materials and Methods

### Substratum Preparation

Tissue culture-treated polystyrene dishes (Corning Life Sciences, Corning, New York) were incubated overnight at 4°C with laminin (Sigma) diluted in PBS. Surfaces were blocked with BSA (Sigma, St. Louis, MO) prior to use.

### Cell Culture

MDCK cells were cultured as described previously [33]. Cells between passage 28 and 36 were utilized. For collision, motility and aggregation assays, confluent MDCK monolayers were suspended using trypsin/EDTA (Invitrogen, Carlsbad, CA) and plated at the desired cell density in serum-free medium (SFM: 1% (v/v) penicillin/streptomycin and 1 mg/mL BSA in Dulbecco’s modified Eagle’s medium (Sigma)) supplemented with 20 ng/mL epidermal growth factor (EGF) (Peprotech, Princeton, NJ). After allowing cells to adhere to the substratum for 1–3 hours, non-adherent cells were washed and the remaining adherent cells were incubated in fresh SFM supplemented with 20 ng/mL EGF.

### Quantification of Cell-cell Adhesion Dynamics

Cell-cell interactions were observed by time-lapse microscopy using a Zeiss Axiovert 200 M microscope with phase contrast images collected every 5 minutes for 24 hours. Two cells were categorized as being in contact if their cell bodies appeared to be touching upon visual inspection of the image. Other published reports have used a similar technique to identify cell-cell contacts [34]. Cell-cell interactions that were initiated in the first 12 hours of observation were tracked, and the duration of cell-cell contact was recorded. Interacting cell pairs that collided with another cell or group of cells were excluded from the analysis.

### Quantification of Migration Speeds

Images of individual migrating cells were acquired every 15 minutes for 15 hours. Cell migration tracks were determined by marking nuclei with ImageJ software (National Institutes of Health, Bethesda, MD), and mean squared displacements were determined for each cell using overlapping intervals [14]. The mean squared displacements were averaged and fit to a persistent random walk model to calculate cell speed, S, and persistence time, P:<d^2^(t)> = 2S^2^P[t−P(1−e^−t/P^)] [13], [14]. Persistence was averaged across all laminin coating concentrations to determine a mean persistence time.

### Quantification of Aggregate Size

Samples were fixed in formalin (Sigma), incubated overnight with glycine in PBS, then permeabilized with 0.2% Triton-X in PBS for 10 minutes at 4 C. Cells were stained with DAPI and the membrane dye FM-464FX (Molecular Probes, Portland, OR). Fluorescence images of 49 non-overlapping fields per condition were captured with a Zeiss Axiovert 200 M microscope and used to determine aggregate sizes with thresholding and edge detection algorithms as previously described [18]. The number of nuclei per field was also quantified in order to calculate the density of substratum-attached cells.

## Supporting Information

Figure S1 Substratum adhesivity affects the fraction of seeded cells that attach to the substratum. 1.5×10^5^ MDCK cells (which is equivalent to 1.6×10^4^ cells/cm^2^) were seeded onto Ln-coated substrata. The fraction of cells that attached to each substratum was quantified after incubation for t_inc_ (1–3 h). Error bars, s.e.m. (n = 3–4 trials).
(TIF)

Figure S2 Substratum adhesivity affects the rate of cell attachment to the substratum. 1.0×10^5^ MDCK cells (which is equivalent to 1.04×10^4^ cells/cm^2^) were seeded onto substrata coated with 0.5 µg/mL (open circles) or 5 µg/mL Ln (closed circles). After incubation for the indicated times, the non-adherent cells were washed, and the number of cells that remained attached was determined. The percent of cells adhered relative to the maximum saturation value is shown (n = 1).
(TIF)

Table S1 Initial density of substratum-attached cells.
(DOC)

Table S2 Incubation times for Ln-coated substrata.
(DOC)

Table S3 Cell seeding concentrations for Ln-coated substrata.
(DOC)

Information S1 **Cell seeding protocol for aggregation experiments.**
(DOC)
