# Supplementary figures and images for: Short-Lived, Transitory Cell-Cell Interactions Foster Migration-Dependent Aggregation
Source: PLoS One. 2012 Aug 17;7(8):e43237. doi: 10.1371/journal.pone.0043237 (PMC3422298; doi:10.1371/journal.pone.0043237)

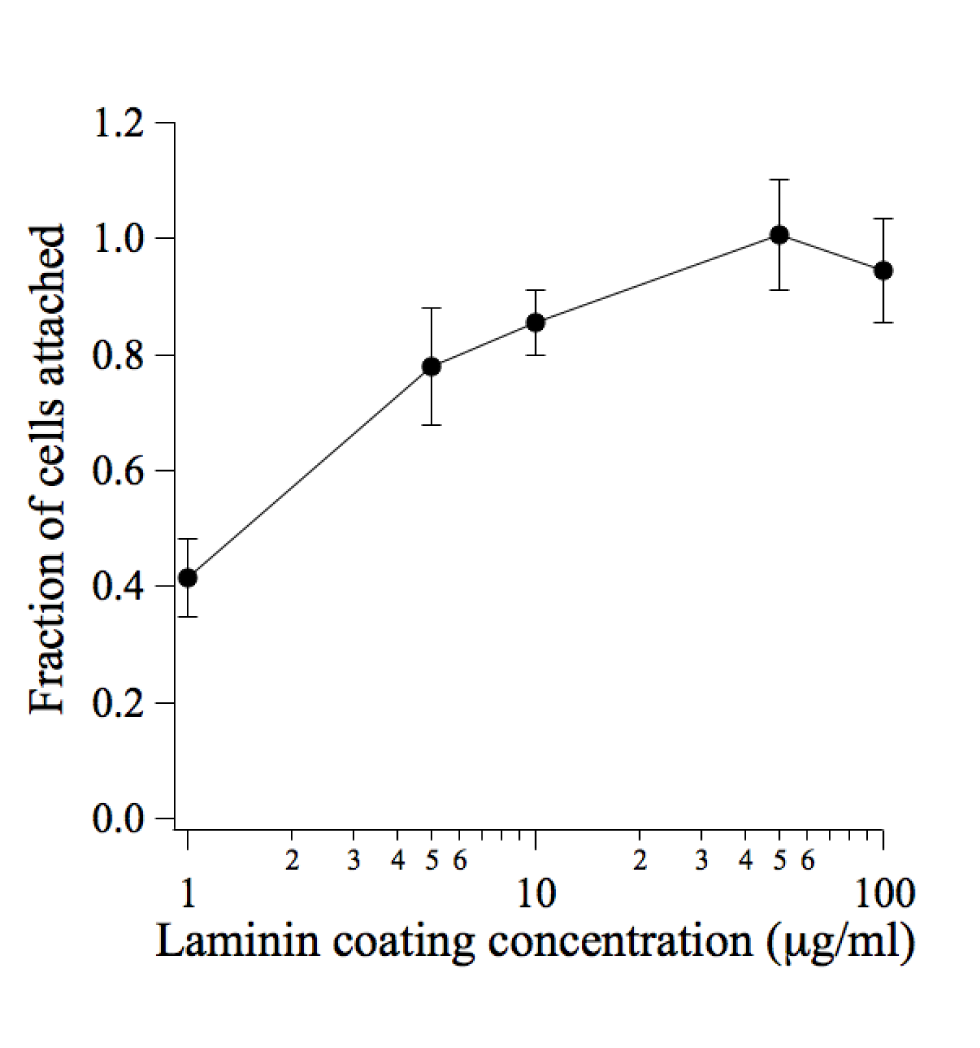

Supplement: Figure S1 — Substratum adhesivity affects the fraction of seeded cells that attach to the substratum. 1.5×10^5^ MDCK cells (which is equivalent to 1.6×10^4^ cells/cm2) were seeded onto Ln-coated substrata. The fraction of cells that attached to each substratum was quantified after incubation for tinc (1–3 h). Error bars, s.e.m. (n = 3–4 trials). (TIF) [file pone.0043237.s001.tif]

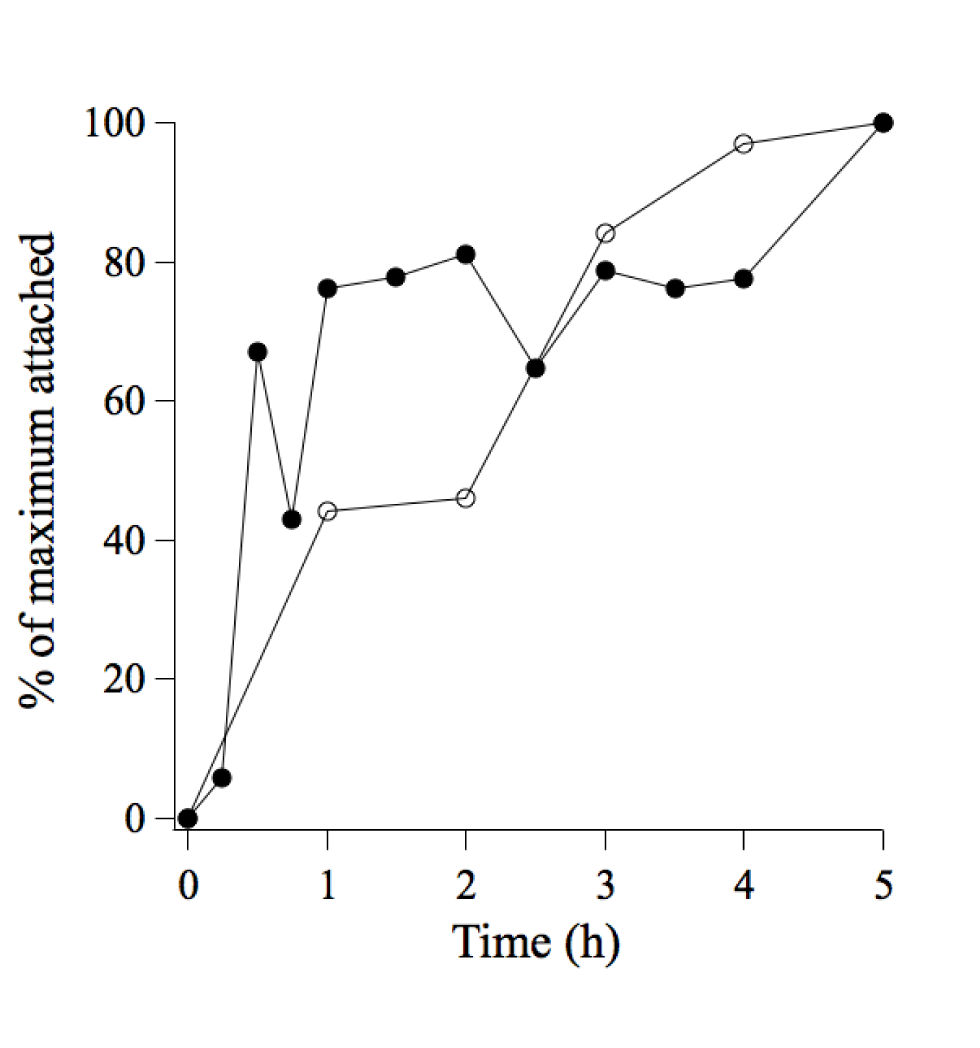

Supplement: Figure S2 — Substratum adhesivity affects the rate of cell attachment to the substratum. 1.0×10^5^ MDCK cells (which is equivalent to 1.04×10^4^ cells/cm2) were seeded onto substrata coated with 0.5 µg/mL (open circles) or 5 µg/mL Ln (closed circles). After incubation for the indicated times, the non-adherent cells were washed, and the number of cells that remained attached was determined. The percent of cells adhered relative to the maximum saturation value is shown (n = 1). (TIF) [file pone.0043237.s002.tif]
