# Supplementary material for: Short-Lived, Transitory Cell-Cell Interactions Foster Migration-Dependent Aggregation
Source: PLoS One. 2012 Aug 17;7(8):e43237. doi: 10.1371/journal.pone.0043237 (PMC3422298; doi:10.1371/journal.pone.0043237)
Supplement: Table S1 — Initial density of substratum-attached cells. (DOC) [file pone.0043237.s003.doc]

**Table S1. Initial density of substratum-attached cells.**

| Ln coating  concentration (μg/mL) | 0.5 | 1 | 5 | 10 | 50 | 100 |
| --- | --- | --- | --- | --- | --- | --- |
| Initial cell surface  density x 10^-3^ (#/cm^2^) | 8.3 ± 0.78 | 8.2 ± 0.32 | 9.1 ± 0.50 | 8.9 ± 0.59 | 8.2 ± 0.59 | 8.2 ± 0.20 |

The number of substratum-attached cells per unit surface area was quantified at initial time (0 h) from fluorescence images of MDCK cells stained with DAPI (n = 3-4 trials).
