# Supplementary material for: Short-Lived, Transitory Cell-Cell Interactions Foster Migration-Dependent Aggregation
Source: PLoS One. 2012 Aug 17;7(8):e43237. doi: 10.1371/journal.pone.0043237 (PMC3422298; doi:10.1371/journal.pone.0043237)
Supplement: Table S2 — Incubation times for Ln-coated substrata. (DOC) [file pone.0043237.s004.doc]

**Table S2. Incubation times for Ln-coated substrata.**

| Ln coating concentration (μg/mL) | 0.5 | 1 | 5 | 10 | 50 | 100 |
| --- | --- | --- | --- | --- | --- | --- |
| t_inc_ (h) | 3 | | 2 | | 1 | |
