# Supplementary material for: Short-Lived, Transitory Cell-Cell Interactions Foster Migration-Dependent Aggregation
Source: PLoS One. 2012 Aug 17;7(8):e43237. doi: 10.1371/journal.pone.0043237 (PMC3422298; doi:10.1371/journal.pone.0043237)
Supplement: Table S3 — Cell seeding concentrations for Ln-coated substrata. (DOC) [file pone.0043237.s005.doc]

**Table S3. Cell seeding concentrations for Ln-coated substrata.**

|  | N_c_ x 10^-4^ (#/mL) | | | | | |
| --- | --- | --- | --- | --- | --- | --- |
| Ln coating concentration (μg/mL) | 3.75 | 5.0 | 6.75 | 7.5 | 10.0 | 12.5 |
| 0.5 |  |  |  | X | X | X |
| 1 |  |  | X | X | X |  |
| 5 |  | X | X | X |  |  |
| 10 |  | X | X | X |  |  |
| 50 | X | X | X |  |  |  |
| 100 | X | X | X |  |  |  |
