# Supplementary material for: Short-Lived, Transitory Cell-Cell Interactions Foster Migration-Dependent Aggregation
Source: PLoS One. 2012 Aug 17;7(8):e43237. doi: 10.1371/journal.pone.0043237 (PMC3422298; doi:10.1371/journal.pone.0043237)
Supplement: Information S1 — Cell seeding protocol for aggregation experiments. (DOC) [file pone.0043237.s006.doc]

**SUPPORTING INFORMATION**

In order to study how local cell-cell reactivity and transport by cell migration affect aggregation dynamics, it was essential to ensure that (1) isolated cells (not pre-formed aggregates) were seeded initially on the substrata, (2) any non-adherent cells were removed to prevent them from drifting and binding to adherent cells/aggregates, and (3) the initial density of isolated cells (and, therefore the initial mean intercellular spacing) was equal among all samples.

To address these considerations, MDCK cells were suspended using traditional cell dissociation techniques, and a desired concentration of single cells (N_c_) was seeded onto Ln-coated substrata. After incubating cells for an appropriate duration (t_inc_) to allow cell adhesion onto the substratum, non-adherent cells were removed by medium aspiration. The key issue, however, was determining the appropriate values for N_c_ and t_inc_ to ensure that the initial cell density on the substratum was equal for all Ln coating concentrations.

We reasoned that cell adhesion to a substratum was likely to proceed more quickly on substrata coated with higher Ln density than on those coated with low amounts of Ln based on reported measurements of cell spreading kinetics on protein-coated surfaces . We quantified the dynamics of cell adhesion in our system (MDCK cells adhering to Ln-coated tissue culture plastic) (Figure S1) and chose an incubation time that allowed cell adhesion to reach its maximum saturation value for each Ln coating concentration (Table S2). This incubation time varied between 1-3 h and was much shorter than the time at which aggregation was quantified (15 h after t_inc_).

In addition to the time of incubation, careful attention was given to the concentration of cells (N_c_) seeded for each Ln coating concentration. We observed that the fraction of seeded cells that attached to the substratum at t_inc_ varied with Ln coating concentration (Figure S2). Therefore, to ensure equivalent initial cell density among the different Ln-coated substrata, we seeded a greater concentration of cells (N_c_) on substrata coated with lower amounts of Ln than on those coated with higher Ln density. Because the fraction of adherent cells was likely to also vary between trials, we took an additional precaution by performing each trial as follows: duplicate sets of cell suspensions containing three closely-spaced N_c_ were seeded for each Ln coating concentration (Table S3). The first set of triplicates was used to determine the seeding concentration that yielded an initial cell density of approximately 8.5 x 10^3^ #/cm^2^. Once identified, the corresponding sample in the second set was used to determine aggregate size at 15 h. In this manner, we ensured that aggregation dynamics were quantified among samples that had an equivalent initial cell density (Table S1).

**REFERENCES**

1. Dubin-Thaler BJ, Giannone G, Dobereiner HG, Sheetz MP (2004) Nanometer analysis of cell spreading on matrix-coated surfaces reveals two distinct cell states and STEPs. Biophys J 86: 1794-1806.
